# Supplementary material for: Cholinergic basal forebrain degeneration due to sleep-disordered breathing exacerbates pathology in a mouse model of Alzheimer’s disease
Source: Nat Commun. 2022 Nov 2;13:6543. doi: 10.1038/s41467-022-33624-y (PMC9630433; doi:10.1038/s41467-022-33624-y)
Supplement: Supplementary file 1 — Supplementary Information [file 41467_2022_33624_MOESM1_ESM.pdf]

Supplementary Material

Cholinergic basal forebrain degeneration due to sleep-disordered breathing exacerbates pathology in a mouse model of Alzheimer's disease.

Lei Qian[1,2,3], Oliver Rawashdeh[3], Leda Kasas[3], Michael R Milne[1,2,3], Nicholas Garner[3], Kornraviya Sankorrakul[3,4], Nicola Marks[1], Matthew W Dean[3], Pu Reum Kim[3], Aanchal Sharma[1], Mark C Bellingham[3], Elizabeth J Coulson [1,2,3*].

1. Queensland Brain Institute,
2. Clem Jones Centre for Ageing Dementia Research,
3. School of Biomedical Sciences, Faculty of Medicine,
   The University of Queensland, Brisbane Qld. 4072, Australia
4. Research Center for Neuroscience, Institute of Molecular Biosciences, Mahidol University, Salaya, Thailand.

* To whom correspondence should be addressed:

Prof. Elizabeth J. Coulson,
School of Biomedical Sciences, The University of Queensland, Brisbane Qld. 4072, Australia. Telephone: +61 7 33653034 Fax: +61 7 33466301 E -mail: e.coulson@uq.edu.au

Supplementary Figure 1

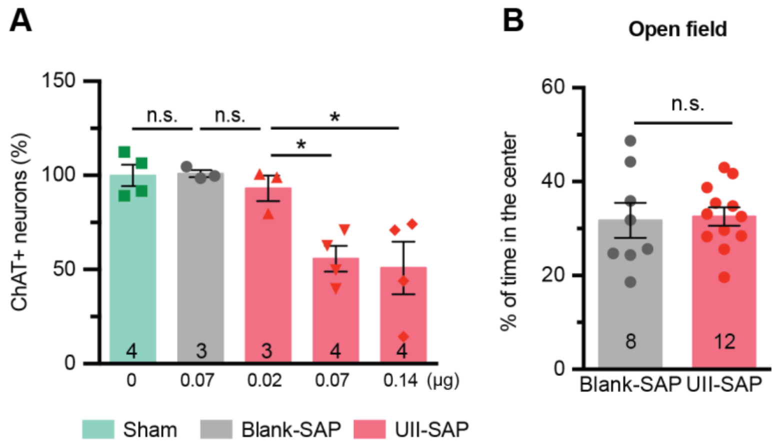

Dose response of UII-SAP-induced cholinergic mesopontine neuronal loss.

Four weeks after direct injection with UII-SAP or control, animals were sacrificed and cholinergic neurons in the mesopontine tegmentum were counted. **(A)** Number of ChAT-positive neurons within the LDT following the direct injection of UII-SAP or IgG-SAP into the MPT at the concentrations shown (P = 0.0015, one-way ANOVA, Tukey's multiple comparison test; Shamvs Blank-SAP: P > 0.9999. **(B)** Time spent in the centre area in the open field test by cMPT- lesioned and sham-lesioned mice (P = 0.5845, Student's unpaired two-tailed t-test). Results are presented as mean ± s.e.m. ** P <0.01 Each data point represents an individual animal.

Supplementary Figure 2 Whole body plethysmographic data of female mice.

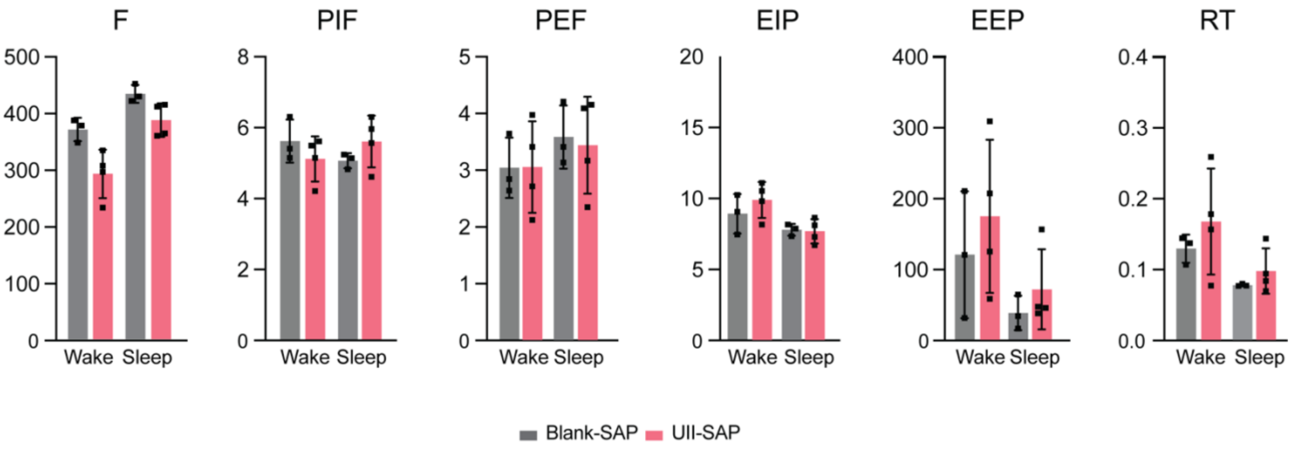

(A) Whole body plethysmography has been used to record respiration during sleep and wake cycles in unrestrained, freely moving female mice. Average polysomnography measures for individual female mice in UII-SAP or control Blank-SAP groups (sleep-wake paired 2 way ANOVA; mean±s.d). Each data point represents an individual animal.

Supplementary Figure 3. EEG-derived sleep-wake metrics of cMPT lesioned mice.

A

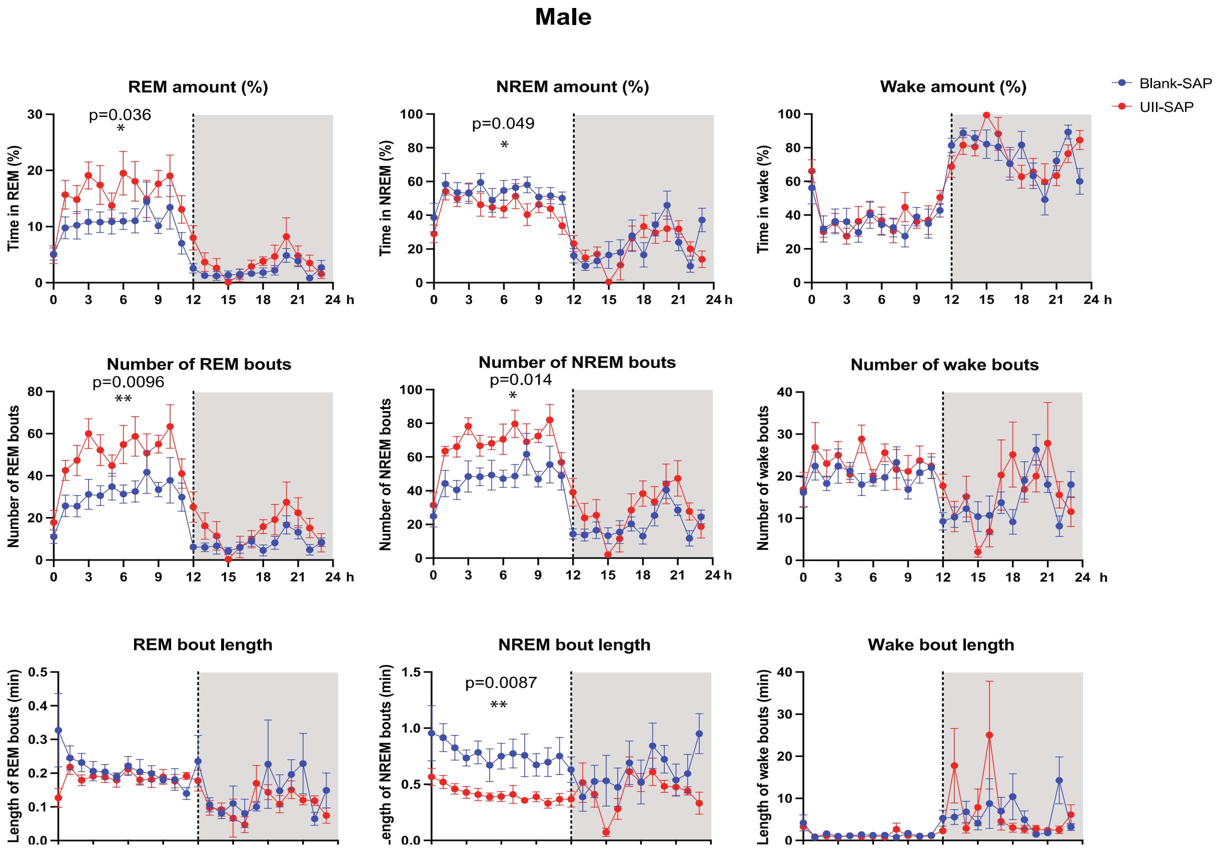

(A) Circadian rhythm of sham-lesion and UII-SAP lesioned male mice

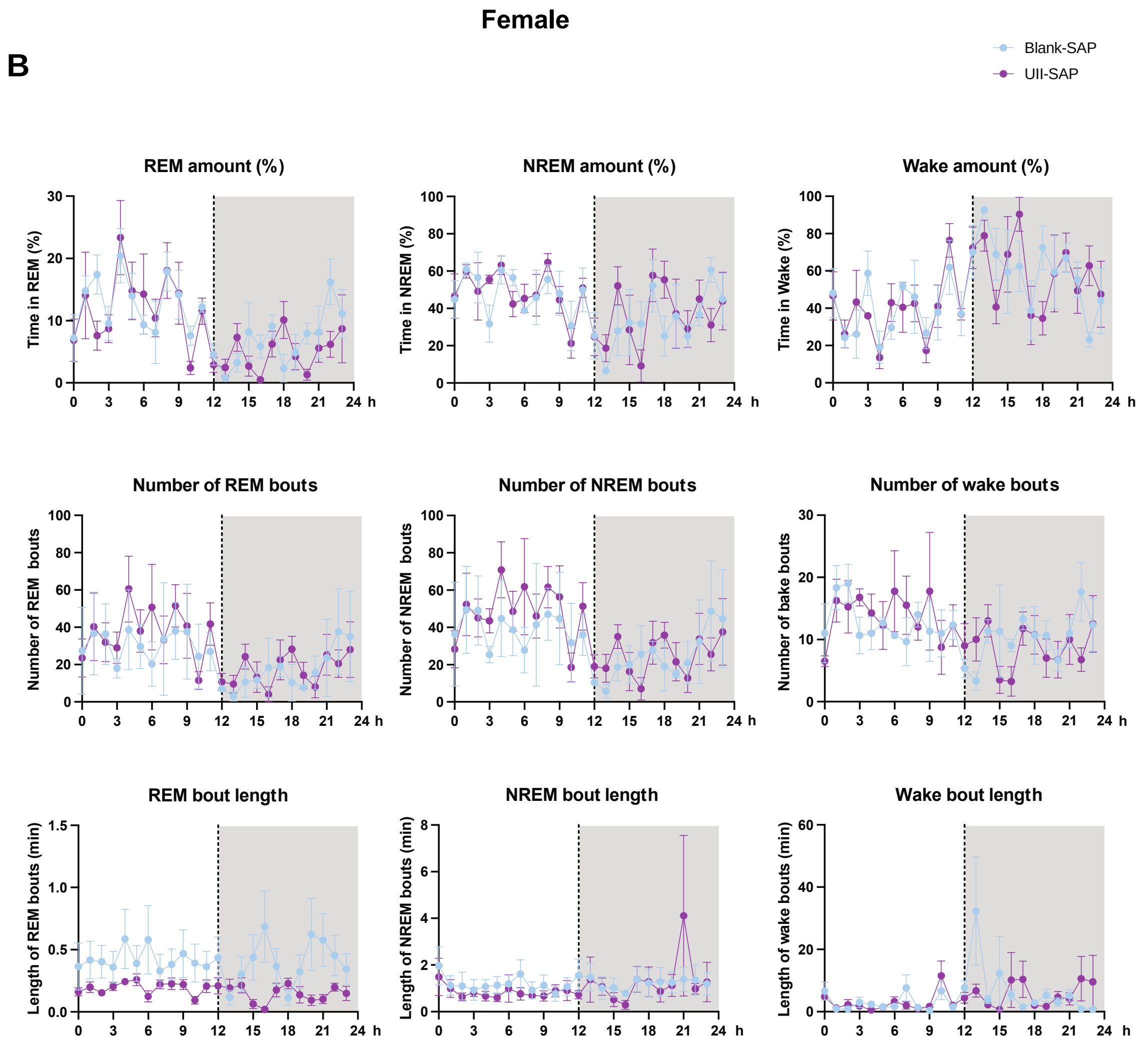

Source of Variation	% of total variation	P value
Time x Lesion	13.58	<0.0001
Time	6.282	0.2325
Lesion	26.02	0.1180
Mouse	36.59	<0.0001

(B) Circadian rhythm of sham-lesion and UII-SAP lesioned female mice

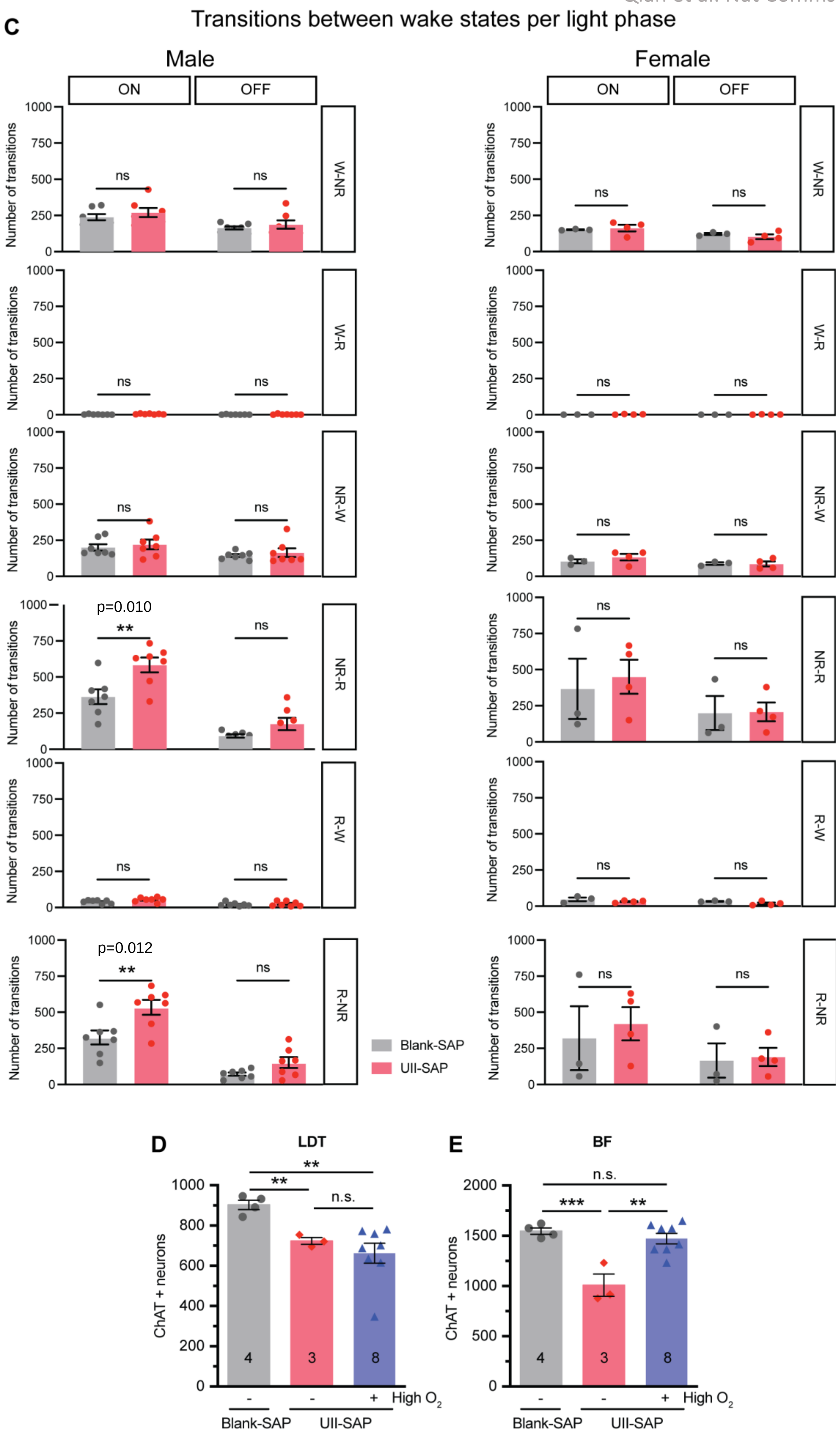
C
Transitions between wake states per light phase
Male
Female
ON
OFF
W-NR
W-R
NR-W
NR-R
R-W
R-NR
Number of transitions
0
250
500
750
1000
ns
p=0.010
**
p=0.012
**
Blank-SAP
UII-SAP
D
LDT
E
BF
ChAT + neurons
0
200
400
600
800
1000
500
1500
2000
**
**
n.s.
***
4
3
8
-
+
High O_2
Blank-SAP
UII-SAP

(D) Total time, number of bouts, bout time and number of transitions between sleep and wake states of sham-lesion and UII-SAP lesioned male and female mice.

(E) Number of LDT neurons in wildtype Blank-SAP- (gray bar) and UII-SAP-injected mice. Both high O_2- (purple bar) and normoxia-treated UII-SAP-injected (red bar) mice had a significant loss of LDT neurons compared to normoxia-treated Blank-SAP-injected mice (*P* = 0.0137, one-way ANOVA, Tukey's multiple comparison test; Blank-SAP vs UII-SAP -O_2: *P* = 0.0021, Blank-SAP vs UII-SAP +O_2: *P* = 0.0089, UII-SAP -O_2 vs +O_2: *P* = 0.4929).

The cBF neuronal number of UII-SAP-injected mice subjected to high oxygen (purple bar) was significantly higher than that of mice subjected to normoxia (red bar), and not significantly different from that of Blank-SAP controls (gray bar), whereas the number of cBF neurons in UII-SAP- injected normoxia-treated mice (red bar) was significantly reduced compared with that of Blank- SAP controls (gray bar) (*P* = 0.0006, Blank-SAP vs UII-SAP -O_2: *P* = 0.0009, Blank-SAP vs UII- SAP +O_2: *P* = 0.6790, UII-SAP -O_2 vs +O_2: *P* = 0.0011).

One way ANOVA with Tukey's multiple comparison test for panels D and E.

Graphs display mean ± s.e.m and each data point represents an individual animal.

Supplementary Figure 4. Histology measure of APP/PS1 mice by sex

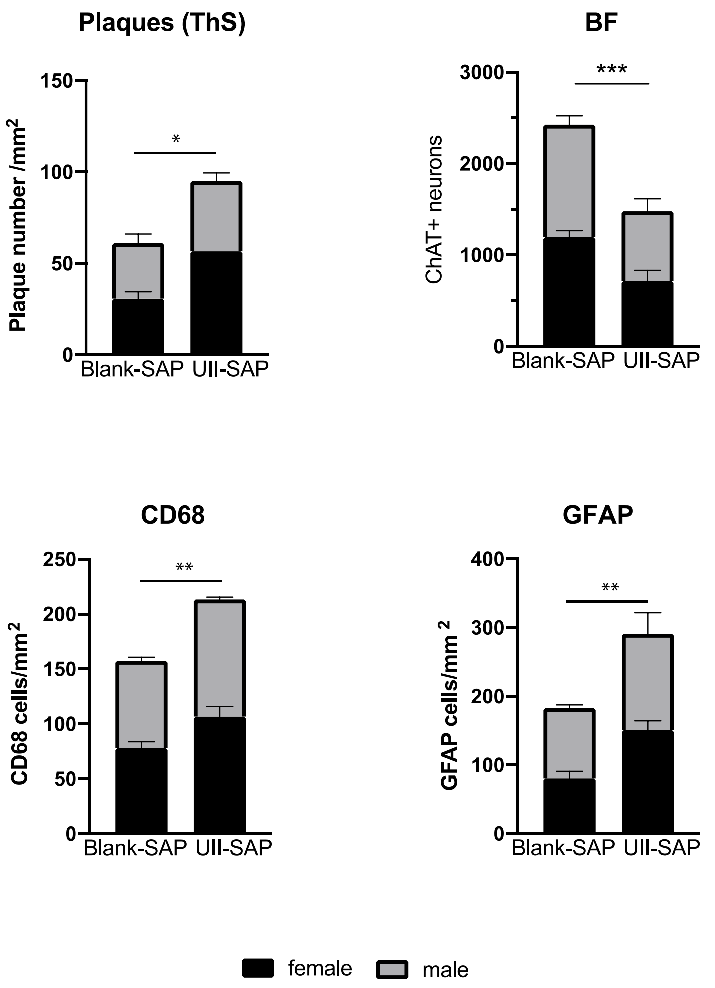

Quantification of thioflavin S-positive plaque number, and ChAT-positive, CD68-positive, and GFAP-positive cell numbers in UII-SAP or control Blank-SAP-injected mice by sex. BF: basal forebrain. No differences in the averages were found by 2 way ANOVA (sex and lesion groups). Graphs display mean ± s.e.m and each data point represents an individual animal.

Supplementary Figure 5. Cholinergic degeneration in the cortex of lesioned APP/PS1 mice

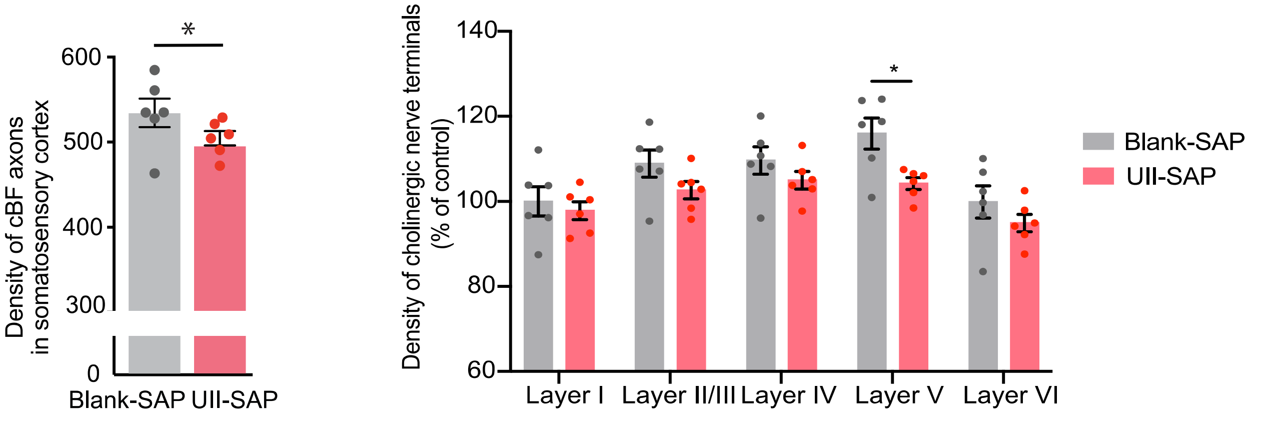

Density of ChAT-positive axons innervating all layers (left; * *P*=0.0131 unpaired two-tailed t test) and each layer (right;* One way ANOVA) of the somatosensory cortex of UII-SAP or Blank-SAP-inject APP/PS1 mice. The graph displays mean ± s.e.m and each data point represents an individual animal.

Supplementary Figure 6. Cholinergic basal forebrain degeneration and behavior following cMPT lesion.

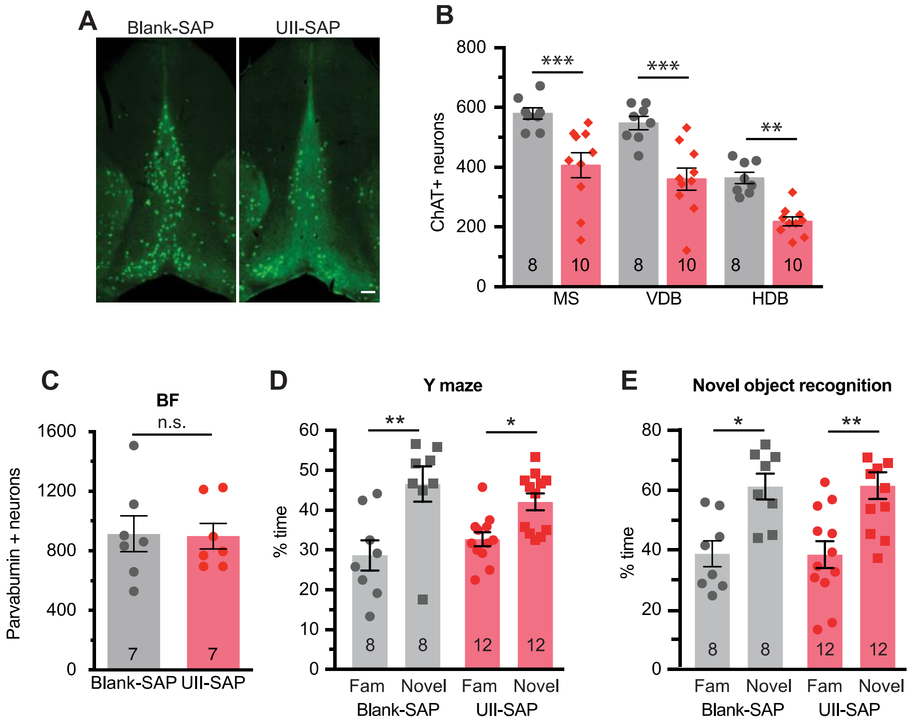

(A) Representative photomicrographs of coronal sections of the basal forebrain immunostained for ChAT-positive neurons following the direct injection of UII-SAP or control Blank-SAP into the MPT (8 animas per group). Scale bar = 200 µm

(B) The number of ChAT-positive neurons in the medial septum (MS), the vertical diagonal bands of Broca (VDB) and the horizontal diagonal bands of Broca (HDB) nuclei of the basal forebrain of mice 8 weeks after injection with UII-SAP or Blank-SAP (two-way ANOVA, Bonferroni's multiple comparisons test; MS: P = 0.0004, VDB: P = 0.0001, HDB: P = 0.0032).

(C) The number of parvalbumin-positive GABAergic neurons in the basal forebrain of mice 8weeks after injection with UII-SAP or Blank-SAP (P = 0.9210, Student's unpaired t-test).

(D) The percentage of time spent in the novel arm of the Y maze on test, compared to the familiar (Fam) arm (P = 0.9493, two-way ANOVA, Tukey's multiple comparison test; Blank-SAP: P =0.0019, UII-SAP: P = 0.0446). Both cMPT-lesioned mice and sham-lesioned wildtype mice displayed a preference for the novel arm.

(E) The percentage of time spent examining a novel object compared to a familiar object (two-way ANOVA, Tukey's multiple comparison test; Blank-SAP: P = 0.0173, UII-SAP: P = 0.0020). Both cMPT-lesioned mice and sham-lesioned mice displayed a preference for the novel object, with no difference in the time spent in each arm between the cMPT-lesioned wildtype mice and sham-lesioned mice.

* $P < 0.05$; ** $P < 0.01$; *** $P < 0.001$; n.s., non-significant. Results are presented as mean ± s.e.m. Each data point represents an individual animal.

Supplementary Figure 7. Lasting effects of the sleep deprivation paradigm.

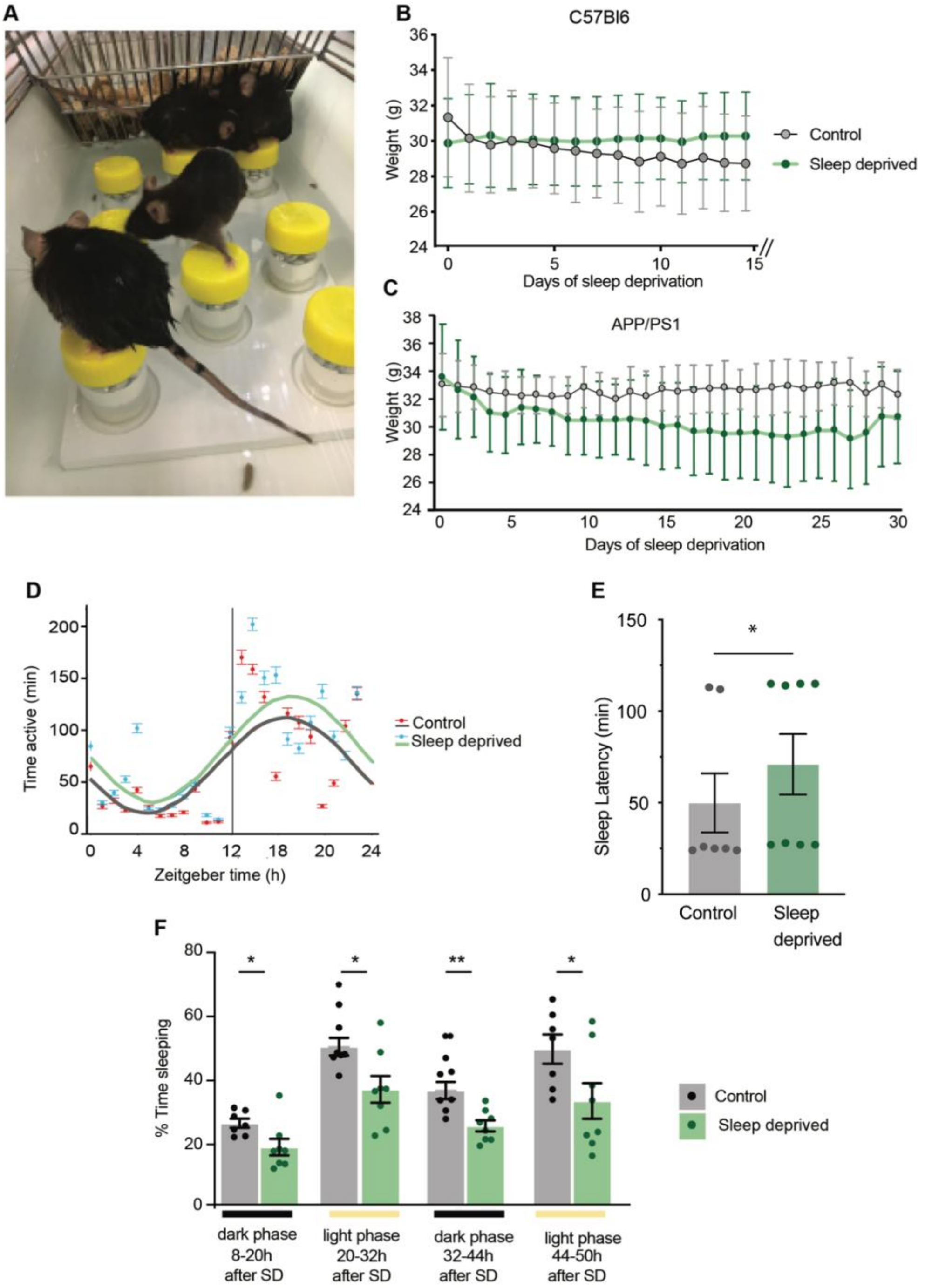

(A) Photograph of the sleep deprivation cages. A video is available in Source Data for Fig 7.

(**B-C**) Weight loss of sleep-deprived and control wildtype **(B)** and APP/PS1 **(C)** mice. mean ± s.d

(D) Sleep-wake rhythm of APP/PS1 mice in the 24 hours immediately following sleep deprivation. mean ± s.e.m **P* < 0.05 ***P* < 0.01; CircaCompare test.

(E) Sleep latency of control and sleep-deprived mice immediately after being transferred from sleep-deprivation cages to home cages on the last sleep-deprivation day. mean ± s.e.m * *P* = 0.0114, unpaired two-tailed Mann-Whitney test.

(F) Sleep analysis of APP/PS1 mice in the 3 days following sleep deprivation. Sleep-deprived mice have reduced sleeptime (*P* values left to right are: 0.0298, 0.0224, 0.0035, 0.0462 [and number of sleep epochs (see Source Data) *P* = 0.0299, 0.224, 0.0035, 0.0303]) in both light and dark periods.

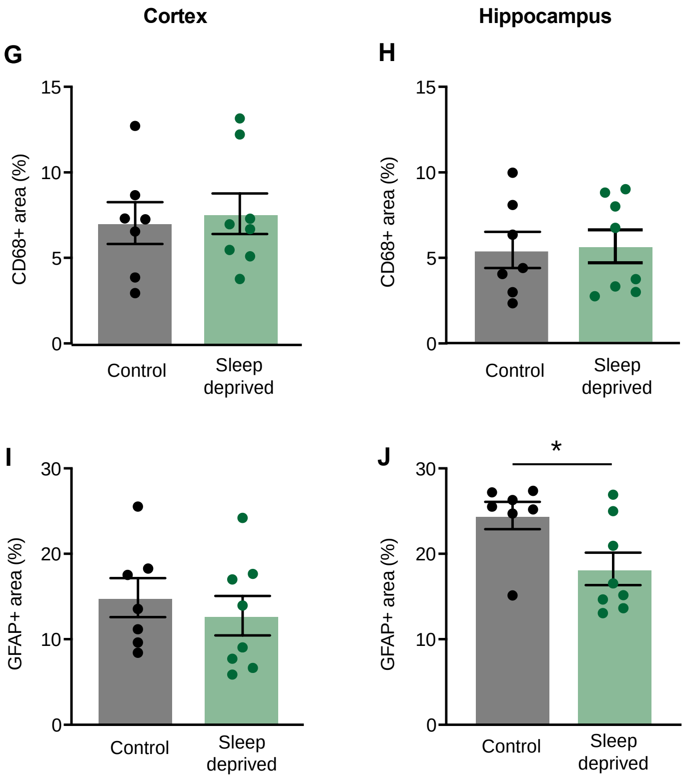

Area of CD68-postive microglia in sensory cortex (**G**) and hippocampus (**H**) of control or sleep-deprived APP/PS1 mice.

Area of GFAP-positive astrocytes in sensory cortex (**I**) and hippocampus (**J**) of control or sleep-deprived APP/PS1 mice. A significant reduction in astrocytosis was found in the hippocampus of sleep-deprived mice compared to controls (* *P*=0.027, unpaired two-tailed t-test).

Results are presented as mean ± s.e.m. Each data point represents an individual animal.

Supplementary Figure 8. 2ME2 treatment protects from OSA-induced cBF degeneration

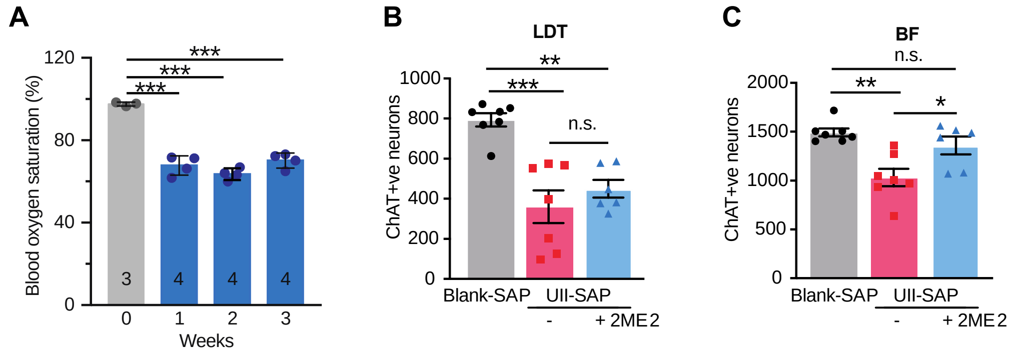

(A) Blood oximetry measurements of mice during each of the 4 weeks of chronic hypoxia exposure.

The number of cMPT **(B)** and cBF **(C)** neurons in a second cohort of mice injected with either Blank-SAP or UII-SAP treated with daily 15mg/kg 2ME2 or vehicle for 27 days (cBF: Blank-SAP vs. UII-SAP: P = 0.0011, Blank-SAP vs. 2ME2 treated: P = 0.020, UII-SAP vs. 2ME2 treated: P =0.4549. As in Fig 9B, 2ME2 treatment protects cBF neurons from the effects of SDB.

*P < 0.05; **P < 0.01; ***P < 0.001; n.s., non-significant, one-way ANOVA with Tukey'smultiple comparison test. Results are presented as mean ± s.e.m. Each data point represents an individual animal.

Supplementary Figure 9. High oxygen rescues APP/PS1 OSA mice from exacerbated AD pathology.

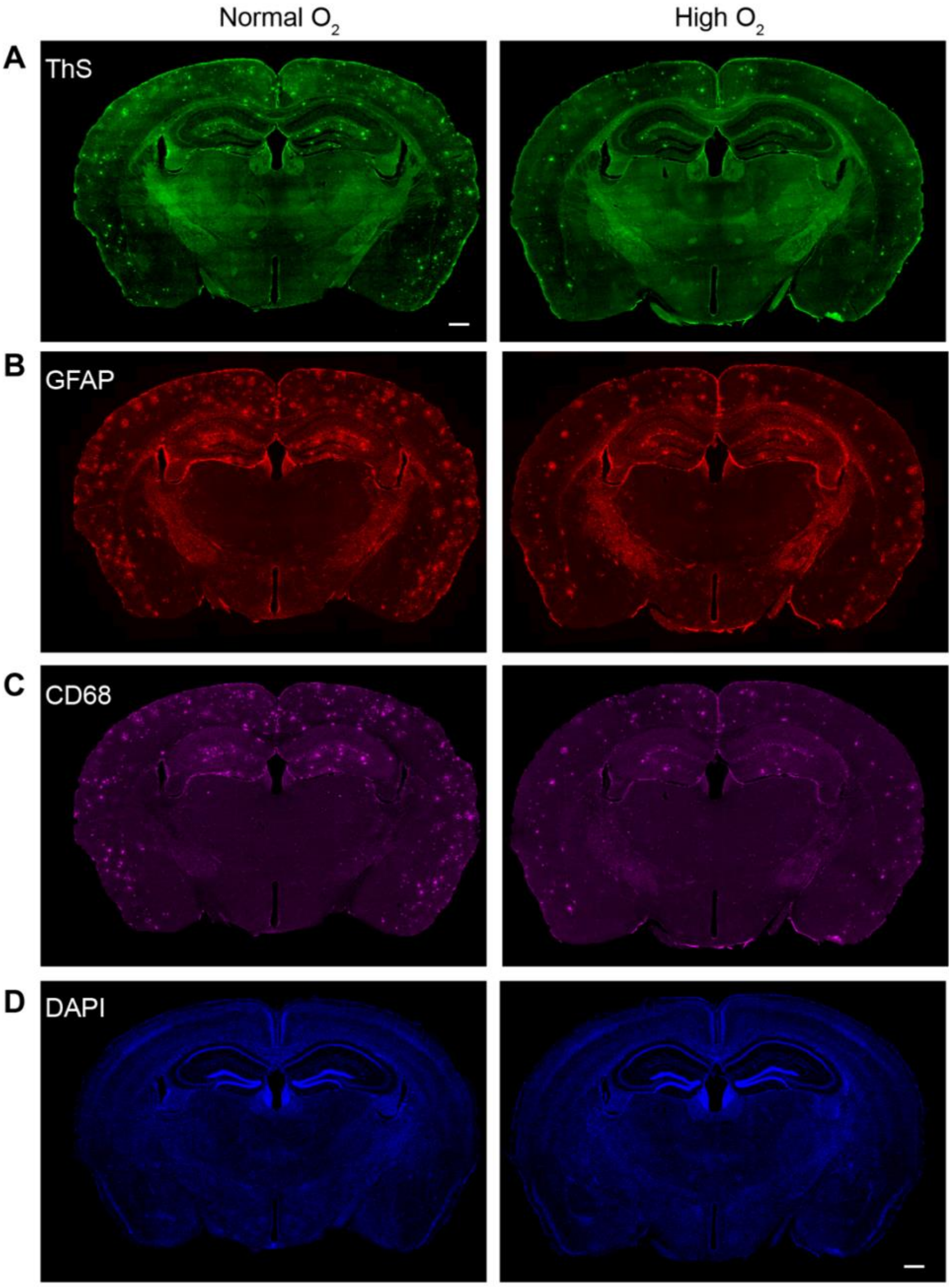

Representative images of **(A)** thioflavin-S (ThS)-positive Aβ plaques, **(B)** GFAP-positive astrocytes, **(C)** CD68-positive microglia, and (**D**) DAPI-positive nuclei in coronal sections containing the neocortex and hippocampus from APP/PS1 mice placed in 40% oxygenated (high O_2, 6 animals) or normoxia (normal O_2, 9 animals) for 8 h a day during the sleep period for 6 weeks, starting 2 weeks after injection with UII-SAP. Scale bars = 200 μm.
