# Supplementary material for: Cholinergic basal forebrain degeneration due to sleep-disordered breathing exacerbates pathology in a mouse model of Alzheimer’s disease
Source: Nat Commun. 2022 Nov 2;13:6543. doi: 10.1038/s41467-022-33624-y (PMC9630433; doi:10.1038/s41467-022-33624-y)
Supplement: Supplementary file 3 — Reporting Summary [file 41467_2022_33624_MOESM3_ESM.pdf]

nature portfolio

Corresponding author(s): Coulson

Last updated by author(s): Aug 17, 2022

Reporting Summary

Nature Portfolio wishes to improve the reproducibility of the work that we publish. This form provides structure for consistency and transparency in reporting. For further information on Nature Portfolio policies, see our Editorial Policies and the Editorial Policy Checklist.

Statistics

For all statistical analyses, confirm that the following items are present in the figure legend, table legend, main text, or Methods section.

n/a	Confirmed	
☐	☒	The exact sample size (n) for each experimental group/condition, given as a discrete number and unit of measurement
☐	☒	A statement on whether measurements were taken from distinct samples or whether the same sample was measured repeatedly
☐	☒	The statistical test(s) used AND whether they are one- or two-sided<br>*Only common tests should be described solely by name; describe more complex techniques in the Methods section.*
☐	☒	A description of all covariates tested
☐	☒	A description of any assumptions or corrections, such as tests of normality and adjustment for multiple comparisons
☐	☒	A full description of the statistical parameters including central tendency (e.g. means) or other basic estimates (e.g. regression coefficient) AND variation (e.g. standard deviation) or associated estimates of uncertainty (e.g. confidence intervals)
☐	☒	For null hypothesis testing, the test statistic (e.g. F, t, r) with confidence intervals, effect sizes, degrees of freedom and P value noted<br>*Give P values as exact values whenever suitable.*
☒	☐	For Bayesian analysis, information on the choice of priors and Markov chain Monte Carlo settings
☒	☐	For hierarchical and complex designs, identification of the appropriate level for tests and full reporting of outcomes
☒	☐	Estimates of effect sizes (e.g. Cohen's d, Pearson's r), indicating how they were calculated

Our web collection on statistics for biologists contains articles on many of the points above.

Software and code

Policy information about availability of computer code

Data collection	TSE PhenoMaster (TSE Systems), Tracker (Bio-Signal Group)v2.1, Zeiss Zen 2012 (Carl Zeiss), SlideBook 6.0 (3I Inc), Nikon NIS(Nikon), Metafer VSlide (MetaSystems), SparkControl (Tecan)
Data analysis	EthoVision XT11 (Noldus Information Technology)v14-15, Imaris 9.2.1 (Bitplane), ImageJ 1.45 (NIH), Prism v7-9 (GraphPad), R v3.

For manuscripts utilizing custom algorithms or software that are central to the research but not yet described in published literature, software must be made available to editors and reviewers. We strongly encourage code deposition in a community repository (e.g. GitHub). See the Nature Portfolio guidelines for submitting code & software for further information.

Data

Policy information about availability of data

All manuscripts must include a data availability statement. This statement should provide the following information, where applicable:

- Accession codes, unique identifiers, or web links for publicly available datasets
- A description of any restrictions on data availability
- For clinical datasets or third party data, please ensure that the statement adheres to our policy

The data that support the findings of this study are available at https://cloudstor.aarnet.edu.au/sender/?s=download&token=bcf92dd8-a16a-430d-a5e1-3442321d9089. (this link will be updated to reflect a published data set with DOI (this will occur once the manuscript is in press ie all data files required are finalized, as the contents can't be changed once it is published) and from the corresponding author.

Human research participants

Policy information about studies involving human research participants and Sex and Gender in Research.

Reporting on sex and gender	n/a
Population characteristics	n/a
Recruitment	n/a
Ethics oversight	n/a

Note that full information on the approval of the study protocol must also be provided in the manuscript.

Field-specific reporting

Please select the one below that is the best fit for your research. If you are not sure, read the appropriate sections before making your selection.

- [x] Life sciences
- [ ] Behavioural & social sciences
- [ ] Ecological, evolutionary & environmental sciences

For a reference copy of the document with all sections, see nature.com/documents/nr-reporting-summary-flat.pdf

Life sciences study design

All studies must disclose on these points even when the disclosure is negative.

Sample size	We generally performed power analyses to estimate sample size based on a pilot study or our previous related data. However, in some situations, the cohorts were determined by he genotypes, age, gender or success of lesion following the surgical procedures.
Data exclusions	The health of the animals following the surgical procedures was resulted in exclusion of animals from subsequent experiments of analyses. Around 7% of Blank-SAP-treated mice and 12% UII-SAP-treated mice were excluded due to health reasons related to the surgical procedure. Where UII-SAP-treated mice did not show >10% cell loss from the mean of control animals they were excluded from the lesion group analyses. Mice showing >3SD from the mean in EEG or breathing measures were excluded from the analysis.
Replication	Numbers of repetitions were clearly indicated in Figure legends or Methods. Results described throughout the paper were reproduced as experimental replicates. Multiple rounds of experiments were performed. Data were acquired from the mice from multiple litters.
Randomization	All the animals were randomly grouped based on the genotype. In mixed gender cohorts, mice of each gender were distributed between groups as equally as possible.
Blinding	For the animal studies, the experiments were recorded using animal ID, without knowledge of the group ID. The grouping information was documented separately, and all the animals were kept in their original cages, rather than being housed in experimental groups. Computer-based automated behavioral analyses ensured unbiased data collection and analysis. For histological analyses of HIF1a staining in nuclei, analyses were performed twice, once by a researcher one blind to any experimental condition and the other who knew the conditions, but not which image was from which condition. Percentages were equivalent.

Reporting for specific materials, systems and methods

We require information from authors about some types of materials, experimental systems and methods used in many studies. Here, indicate whether each material, system or method listed is relevant to your study. If you are not sure if a list item applies to your research, read the appropriate section before selecting a response.

Materials & experimental systems

n/a	Involved in the study	
[ ]	[x]	Antibodies
[x]	[ ]	Eukaryotic cell lines
[x]	[ ]	Palaeontology and archaeology
[ ]	[x]	Animals and other organisms
[x]	[ ]	Clinical data
[x]	[ ]	Dual use research of concern

Methods

n/a	Involved in the study	
[x]	[ ]	ChIP-seq
[x]	[ ]	Flow cytometry
[x]	[ ]	MRI-based neuroimaging

Antibodies

Antibodies used	Goat anti-ChAT (Millipore, AB144P),<br>Mouse anti-parvalbumin (Millipore, MAB1572),<br>Rabbit anti-calbindin (Swant, CB38),<br>Mouse anti-Aβ (6E10, Convance, Sig-39320),<br>Rabbit anti-GFAP (Dako, Z0334),<br>Rat anti-CD68 (FA-11, AbD Serotec, MCA1957),<br>Rabbit ant-HIF1α (Novus Biologicals, NB100-479),<br>Donkey anti rabbit AlexaFluor647 (Invitrogen, A31573),<br>Donkey anti-rabbit AlexaFluor 594 (A21207), Donkey anti-rabbit AlexaFluor488 (A21206), Donkey anti-mouse AlexaFluor 647 (A31571), Donkey anti-mouse AlexaFluor 488 (A21202), Donkey anti-goat AlexaFluor647 (A21447), Donkey anti-goat AlexaFluor 488 (A11055), and Donkey anti-rat AlexaFluor 647 are all from Jackson ImmunoResearch, 712-605-153.
Validation	All primary antibodies are widely used commercially available antibodies. The antibodies were validated in IHC by the manufacturer or by previously published studies.<br>anti-ChAT (Millipore, PMID: 25602013), anti-parvalbumin (Millipore, PMID: 25599221), anti-calbindin (Swant, PMID: 30078578), anti-Aβ (Convance, PMID: 19246392), anti-GFAP (Dako, PMID: 31953387), anti-CD68 (AbD Serotec, PMID: 31316211), ant-HIF1α (Novus Biologicals, PMID: 19336759).

Animals and other research organisms

Policy information about studies involving animals; ARRIVE guidelines recommended for reporting animal research, and Sex and Gender in Research

Laboratory animals	Mice of either sex were used, unless indicated. The age of the mice are clearly indicated in the manuscript.<br>C57BL/6j, Jackson Laboratory: 000664 (Aged 8 weeks to 6 months). APP/PS1, Jackson Laboratory: 34832 (Aged 8 month to 16 months). ChAT-IRES-Cre, Jackson Laboratory: 006410 (aged 8 weeks to 6 months); Hif1a floxed, Jackson Laboratory 007561 (aged 8 weeks to 6 months).
Wild animals	This study did not involve wild animals.
Reporting on sex	The gender of the animals is provided for each data set.
Field-collected samples	This study did not involve wild animals.
Ethics oversight	All procedures were approved by the University of Queensland Animal Ethics Committee which was conducted in accordance with the Australian Code of Practice for the Care and Use of Animals for Scientific Purposes (8th edition, 2013).

Note that full information on the approval of the study protocol must also be provided in the manuscript.
